# Nivalenol affects Cyclin B1 level and activates SAC for cell cycle progression in mouse oocyte meiosis

**DOI:** 10.1111/cpr.13277

**Published:** 2022-06-23

**Authors:** Ping‐Shuang Lu, Yue Wang, Le Jiao, Shao‐Chen Sun

**Affiliations:** ^1^ College of Animal Science and Technology Nanjing Agricultural University Nanjing China

## Abstract

**Objectives:**

Nivalenol (NIV) is a secondary metabolite of type B trichothecene mycotoxin produced by *Fusarium* genera, which is widely found in contaminated food and crops such as corn, wheat and peanuts. NIV is reported to have hepatotoxicity, immunotoxicity, genotoxicity, and reproductive toxicity. Previous studies indicate that NIV disturbs mammalian oocyte maturation. Here, we reported that delayed cell cycle progression might be the reason for oocyte maturation defect caused by NIV exposure.

**Methods and Results:**

We set up a NIV exposure model and showed that NIV did not affect G2/M transition for meiosis resumption, but disrupted the polar body extrusion of oocytes. Further analysis revealed that oocytes were arrested at metaphase I, which might be due to the lower expression of Cyclin B1 after NIV exposure. After cold treatment, the microtubules were disassembled in the NIV‐exposed oocytes, indicating that NIV disrupted microtubule stability. Moreover, NIV affected the attachment between kinetochore and microtubules, which further induced the activation of MAD2/BUBR1 at the kinetochores, suggesting that spindle assemble checkpoint (SAC) was continuously activated during oocyte meiotic maturation.

**Conclusions:**

Taken together, our study demonstrated that exposure to NIV affected Cyclin B1 expression and activated microtubule stability‐dependent SAC to ultimately disturb cell cycle progression in mouse oocyte meiosis.

## Graphical abstract

The image showed that NIV exposure caused the microtubule instability, which disrupted the kinetochore‐microtubule attachment, and further activated spindle assembly checkpoint in mouse oocytes. Green, α‐tubulin; Blue, DNA.

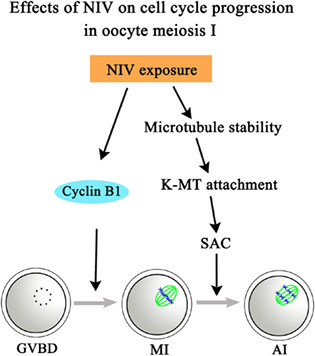

**Abbreviations**
GVBD: germinal vesicle breakdown
K‐MT: kinetochore microtubule attachment
MI: metaphase I
MII: metaphase II
NIV: Nivalenol
PB1: first polar body

## 1 INTRODUCTION

Food safety is a global concern especially in the situation of high mycotoxin contamination occurrence.^1^ About 25% of the crops in the world have been contaminated by molds and fungi growth, which may further aggravate under the on‐going global warming.^2^ Mycotoxins are a group of low molecular compounds with structural diversity produced by different types of fungi.^3^ One of the most common fungi found worldwide is *Fusarium* genera.^4^ Nivalenol (NIV) is considered to be an important secondary metabolite of trichothecene mycotoxin produced by *Fusarium* species since it is frequently found in contaminated crops such as corn, wheat and peanuts.^5^ Previous studies have demonstrated that NIV is obviously toxic to digestive and immune systems. Animal experiments show that NIV not only significantly decreases red and white blood cell count, but also affects the lymph follicle germ centers of spleen, lymph node, thymus and bone marrow in mice.^6^, ^7^ NIV also accelerates J774A.1 macrophages apoptosis and shows anti‐proliferative effects with a dose‐dependent manner.^8^ In addition, 0.5 μg/ml NIV induces HeLa cells G1/S and G2/M transition failure by means of autoradiography.^9^ Recent evidences have identified that NIV is also potentially toxic to reproductive system. In males, testis damage and reduction on spermatogenic cells number are observed after NIV exposure.^10^ In females, subchronic toxicity study has suggested that 100 ppm NIV increases ovarian follicular atresia and inhibits corpus luteum development in F344 rats.^11^ Besides, our previous results show that NIV disrupts mitochondria function and induces apoptosis during oocyte maturation.^12^, ^13^

Oocyte meiotic maturation is a prerequisite for successful fertilization and subsequent embryo development. Meiosis resumption (G2/M transition) and the progression to metaphase II (MII) are two major stages in oocyte meiosis.^14^ Oocyte is arrested in germinal vesicle (GV) stage until luteinizing hormone (LH) surge stimulation, quickly followed by germinal vesicle breakdown (GVBD) which marks the beginning of prometaphase I (pro‐MI).^15^ After oocyte enters pro‐MI, acentriolar microtubule organizing centers (MTOCs) near the nuclear envelope are fragmented and highly dynamic microtubules capture the condensed chromosome to build attachment between kinetochore and microtubules (K‐MT), indicating that the initiation of bipolar spindle formation and chromosome transport to the cell equator.^16^ The microtubules which encounter and build correct attachment with a kinetochore become stabilized, whereas are depolymerized soon.^17^ Then oocyte reaches metaphase I (MI) when chromosomes align at the spindle equator. At MI stage, the spindle migrates to the oocyte cortex along its long axis, waiting the signal of metaphase‐anaphase transition to progress through MI and extrude the first polar body (PB1).^18^ Finally, oocyte is arrested at MII stage until fertilization.

The successful cell cycle progression of meiosis I is depended on high metaphase promoting factor (MPF) activity.^19^ MPF, a complex of Cyclin B1 and cyclin‐dependent kinase (CDK1), shows its catalytic activity though CDK1 and it is noted that binding to Cyclin B1 is essential for CDK1 activation.^20^ When all chromosomes build stable attachment with microtubules and align at the spindle plate, Cyclin B1 is dramatically degraded by an APC/C‐dependent process and subsequently inactivate MPF.^21^ Besides, proper anaphase onset and chromosome segregation are also critical to ensure oocyte maturation quality. Correct and stable bipolar K‐MT attachment is monitored by the meiotic spindle‐assembly checkpoint (SAC) which could produce a “wait‐anaphase” signal to delay anaphase for faithful chromosome segregation during oocyte maturation.^17^ Several core components of SAC proteins, such as MAD1, MAD2, BUB1, BUB2, and BUBR1 (MAD3 in yeast) are reported to regulate metaphase I arrest in meiosis.^22^ It is suggested that unattached kinetochores can recruit MAD2 to bind CDC20, the co‐activator of APC/C, and further associate with BUBR1 and BUB3 to form the mitotic checkpoint complex (MCC) for cell cycle control.^23^

Although the toxicity of NIV on oocyte and sperm are reported, the mechanism is still unclear. In this study, we investigated the effects of NIV on cell cycle control during oocyte meiotic maturation. Surprisingly, our results showed that NIV did not affect G2/M transition; however, a significant reduction in Cyclin B1 level, the disrupted microtubule stability and K‐MT attachment indicated that NIV induced metaphase I arrest in mouse oocytes. Our data provided an explanation for the NIV effects on oocyte maturation quality from cell cycle aspect.

## 2 MATERIALS AND METHODS

### 2.1 Antibodies and chemicals

Rabbit polyclonal anti‐gamma H2AX antibody (ab2893), rabbit monoclonal anti‐Cyclin B1 antibody (ab181593), mouse monoclonal anti‐CDK1 antibody (ab18), and sheep polyclonal anti‐BUBR1 antibody (ab28193) were from Abcam (Cambridge, UK). Rabbit polyclonal anti‐MAD2 antibody (10337‐1‐AP) and CoraLite594‐conjugated donkey anti‐Rabbit IgG (H + L) (SA00013‐8) were from Proteintech (Wuhan, China). Alexa Fluor 594 goat anti‐rabbit antibody, Alexa Fluor 488, and 594 goat anti‐sheep antibody were from Invitrogen (Carlsbad, CA). Rabbit polyclonal anti‐centromere (15‐234‐0001) was from Antibodies Incorporated (Shenzhen, China). Horseradish peroxidase‐conjugated goat anti‐rabbit/mouse IgG antibodies were from Beyotime (Nantong, China). Mouse monoclonal anti‐α‐tubulin‐FITC antibody (F2168), Hoechst 33342 (B2261) and all other unstated chemicals were from Sigma (St. Louis, MO).

### 2.2 Oocyte collection and culture

All operations on mice were followed the guidelines of the Animal Research Committee of Nanjing Agricultural University, China (XYXK‐Su‐2017‐0007). The experiments were specifically approved by the committee of Animal Ethics and Farewell in Nanjing Agricultural University. Institute of Cancer Research (ICR) female mice under 4 to 6 weeks of age were used in this study. Denuded germinal vesicle stage oocytes were obtained from chopped ovaries with a prefabricated glass tube and then cultured in M16 medium under liquid paraffin oil at 37 °C in 5% CO_2_ atmosphere for specific times.

### 2.3 Nivalenol treatment

The nivalenol (NIV) was dissolved in dimethyl sulfoxide (DMSO) to a 50 mM reserve solution. For high‐dose groups, 50 mM NIV was directly diluted to final concentration of 100 and 200 μM with M16 medium. For low dose groups, 50 mM NIV was first diluted to a median concentration of 2 mM with DMSO and then produced the final concentrations of 3 and 5 μM in M16 medium, which was based on previous studies on oocytes.^24^ The final DMSO concentration administered to oocytes was <0.4%. For release groups, we washed the oocytes 10 times (2 min each) in fresh M2 medium after 2 h culture with 5 μM NIV, the time point when most oocytes underwent GVBD. Then these oocytes were transferred to fresh M16 medium and cultured another 10 h under liquid paraffin oil at 37 °C in 5% CO_2_ atmosphere, since most oocytes reached MII stage after 12 h culture.

### 2.4 Immunofluorescence staining and confocal microscopy

For detections of γ‐H2AX, Cyclin B1, α‐tubulin and MAD2, the oocytes were fixed in 4% paraformaldehyde for 30 min at room temperature, followed by permeabilization with 0.5% Triton X‐100 for 20 min and blocking in 1% BSA‐supplemented phosphate buffer saline (PBS) for 1 h. For kinetochore immunostaining and cold‐stable microtubules visualization, M2 medium was pre‐cooled at 4 °C for at least 30 min and oocytes were transferred to the medium for 5 min cold treatment before fixation. For BUBR1 staining, oocytes were first transferred to 0.5% Triton X‐100 for 5 min and then washed three times (5 min each) in washing buffer (0.1% Tween 20 and 0.01% Triton X‐100 in PBS). After fixation with 4% paraformaldehyde for 20 min at room temperature and three washes (5 min each) in washing buffer, oocytes were blocked 1 h in 1% BSA (in PBS). Then the samples were incubated with primary antibodies for 3 h at room temperature or overnight at 4 °C and subsequently washed three times in washing buffer before labeled with the corresponding secondary antibodies for 1 h at room temperature. The primary antibodies used were anti‐γ‐H2AX (1:100), anti‐Cyclin B1 (1:800), anti‐α‐tubulin‐FITC (1:200), anti‐MAD2 (1:100), anti‐centrosome (1:100), and anti‐BUBR1 (1:50). The secondary antibodies used were Alexa Fluor 488/594 goat anti‐rabbit or sheep IgG (1:200) and 594 donkey anti‐Rabbit IgG (1:800). Three washes in washing buffer after that, the oocytes were then stained with Hoechst 33342 for 15 min at room temperature and mounted on glass slides for examination by a confocal laser scanning microscope (Zeiss LSM 800 META, Jena, Germany).

### 2.5 Fluorescence intensity analysis

Image J software (National Institutes of Health, Bethesda, MD) was used for fluorescence intensity measurement. The control and treated oocytes were placed in different area on the same glass slide and scanned in the same environment using the same parameters. A region of interest on the target image was detected and the average of all mean values between the control and treated groups were used to perform statistical analysis.

### 2.6 Western blot analysis

A total of 150 oocytes per group were lysed with NuPAGE LDS Sample Buffer and boiled at 100 °C for 10 min. The proteins were separated by electrophoresis on 10% Sodium dodecyl sulfate‐polyacrylamide gel electrophoresis (SDS‐PAGE) at 160 V for 80 min, followed by transferring onto polyvinylidene fluoride membranes (Millipore, Billerica, MA) at 20 V for 1 h. Then the membranes were blocked with TBST containing 5% nonfat milk for at least 2 h at room temperature and subsequently incubated with anti‐β‐actin (1:1000), anti‐CDK1 (1:1000), and anti‐Cyclin B1 (1:2000) at 4 °C overnight. After three washes in TBST (10 min each), immunoblots were labeled with conjugated anti‐mouse or anti‐rabbit antibodies (1:1000) for 1 h at room temperature. Finally, three washes in TBST were performed and the membranes were processed using the ECL Plus Western Blotting Detection System (Tanon‐3900, China) and then Image J software was used to analyze the band intensity values.

### 2.7 Statistical analysis

At least three independent biological replicates were performed for each treatment. Statistical comparisons were conducted by paired‐wise *t*‐test or ANOVA using GraphPad Prism 5 software (GraphPad, San Diego, CA). Data were expressed as mean ± SEM and the number of oocytes observed (*n*) was put in parentheses. *p* < 0.05 was considered statistically significant.

## 3 RESULTS

### 3.1 NIV does not affect G2/M transition in oocyte meiosis

**FIGURE 1:**
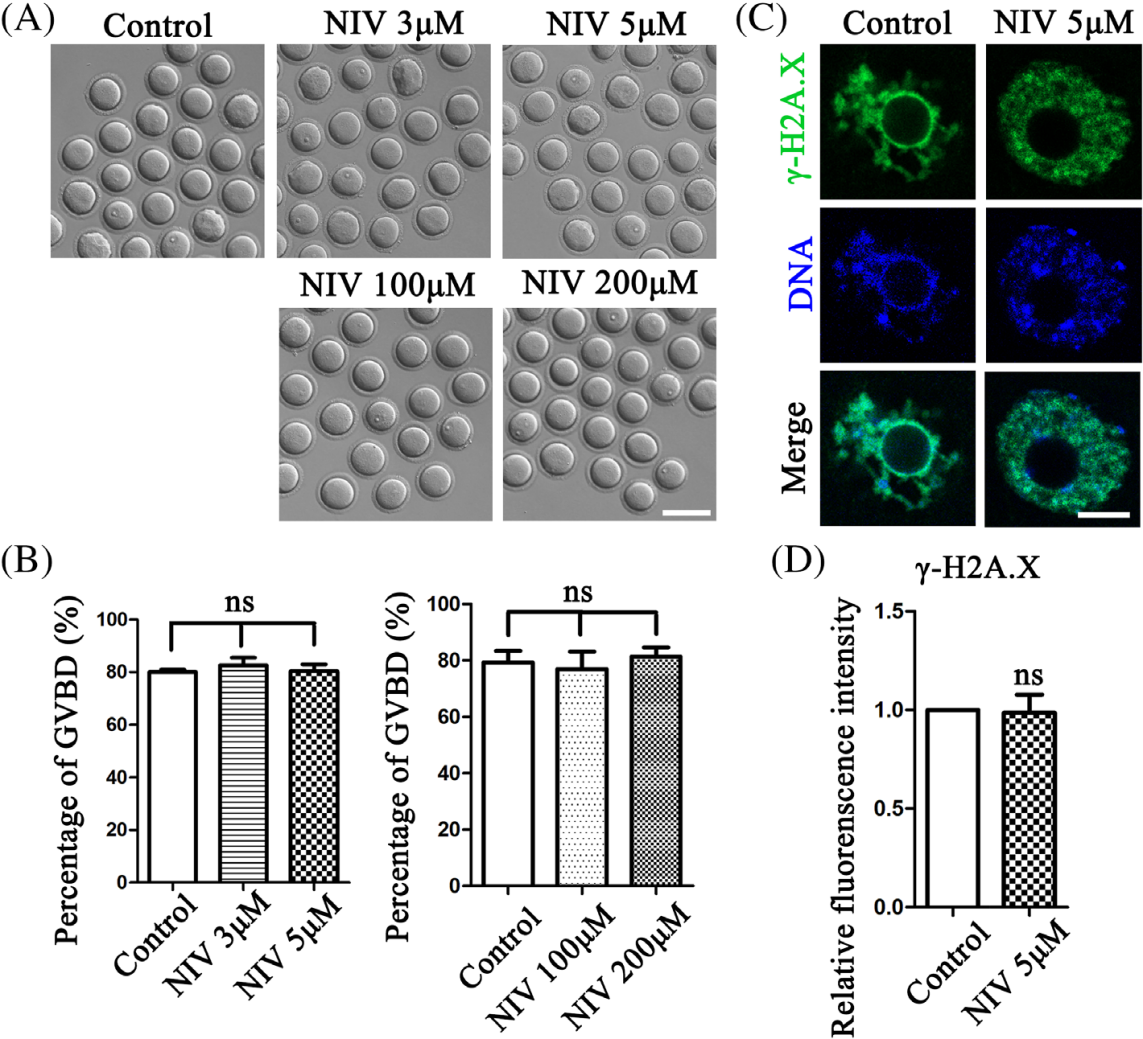
NIV does not affect G2/M transition in oocyte meiosis. (A) The typical image of oocytes underwent GVBD after 2 h culture in the low and high does NIV treatment groups. (Control: *n* = 121; 3 μM group: *n* = 118; 5 μM group: *n* = 116; 100 μM group: *n* = 113; 200 μM group: *n* = 124). Bar = 100 μm (B) The GVBD rate of NIV treatment groups was similar with control groups. ns, *p* > 0.05. (C) Comparing with the control group, there was no change in the 5 μM NIV treatment group after 50 min culture (Control: *n* = 48; 5 μM group: *n* = 52). Green, γ‐H2AX; Blue, DNA; Bar = 5 μm. (D) The fluorescence intensity of γ‐H2AX showed no significant difference between control and 5 μM NIV‐treated groups. ns, *p* > 0.05.

We first examined the effects of NIV on meiosis resumption in mouse oocytes. As shown in Figure 1A, there were no effects of NIV on GVBD of mouse oocytes after culturing 2 h, which could be confirmed by the ratio analysis (Control: 80.00 ± 1.00%, *n* = 121; 3 μM: 82.67 ± 2.91%, *n* = 118, *p* > 0.05; 5 μM: 80.33 ± 2.60%, *n* = 116, *p* > 0.05; Figure 1B). Similar results were found even we tried high doses of NIV exposure with 100 and 200 μM concentrations. The statistical analysis data for the percentage of GVBD after NIV treatment also confirmed this, showing with no significant difference for the ratio between the control and treated groups (Control: 79.33 ± 4.10%, *n* = 116; 100 μM: 77.00 ± 6.24%, *n* = 113, *p* > 0.05; 200 μM: 81.33 ± 3.38%, *n* = 124, *p* > 0.05; Figure 1B). We selected 5 μM for consequent experiments.

DNA damage could activate G2/M checkpoint and cause the arrest of oocytes at GV stage. We then used DNA damage marker γ‐H2AX labeling after culturing 50 min to confirm the effects of NIV on meiosis resumption. Our results showed that the γ‐H2AX fluorescent signals of control group had no significant difference with 5 μM NIV‐treated group (Figure 1C). And the fluorescence intensity analysis also confirmed this (Control: 1.00 ± 0.00, *n* = 48; 5 μM: 0.99 ± 0.09, *n* = 52, *p* > 0.05; Figure 1D), indicating that exposure to NIV did not induce DNA damage and following G2/M transition failure in GV oocytes.

### 3.2 NIV causes metaphase I arrest in oocyte meiosis

**FIGURE 2:**
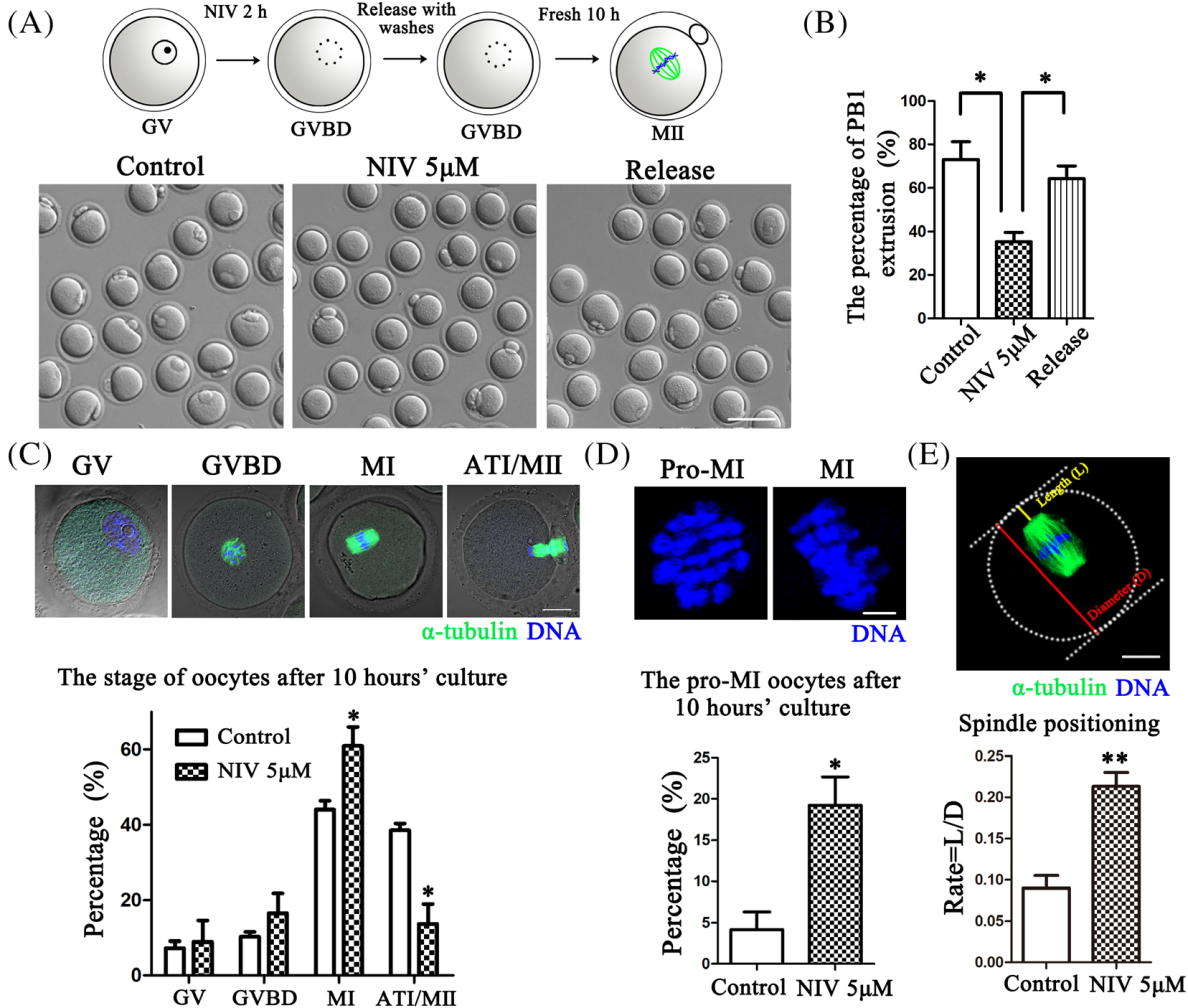
NIV causes metaphase I arrest in oocyte meiosis. (A) Scheme for the release approach after NIV exposure and the typical image for the PB1 extrusion of control, NIV‐treated and 2 h following release from 5 μM NIV groups (Control: *n* = 130; NIV group: *n* = 121; NIV release group: *n* = 126). Bar = 100 μm. (B) The rate of PB1 extrusion was significantly decreased in the NIV‐treated groups compared with the control and NIV‐release groups. *p < 0.05. (C) Different stages of oocytes during meiosis after NIV treatment (Control: *n* = 166; NIV group: *n* = 137). Green: α‐tubulin; Blue: DNA; Bar = 20 μm. The proportion of ATI/MII stage was much lower but higher for MI stage after NIV treatment. *p < 0.05. (D) The rate of pro‐MI stage oocyte was much higher after exposure to NIV than the control group. Blue, DNA; Bar = 5 μm; *p < 0.05. (E) Measurement of spindle position to cortex. We defined the distance from the spindle pole to cortex as L and the dimeter of oocytes as D. The ratio of L/D was considerably increased after NIV exposure (Control: *n* = 43; NIV group: *n* = 47). Green, α‐tubulin; Blue, DNA; Bar = 20 μm; **p < 0.01.

We next adopted NIV release approach to check the effects of NIV on oocyte cytokinesis. As shown in Figure 2A, the polar body extrusion of NIV‐treated oocytes after 12 h culture was affected; however, when we released and washed the oocytes after 2 h of NIV culture, the oocytes resumed to reach MII after 10 h culture in fresh medium. And statistical analysis data for the percentage of polar body extrusion showed that it was significant higher in release group compared with the NIV‐exposed group (Control: 73.11 ± 8.06%, *n* = 130; NIV‐treated group: 35.26 ± 4.35%, *n* = 121, *p* < 0.05; NIV‐released group: 64.35 ± 5.79%, *n* = 126, *p* < 0.05; Figure 2B), indicating that NIV exposure affected the cell cycle progression in mouse oocytes.

We then examined the proportion of oocytes in different cell cycle stages during meiotic maturation by staining microtubule and chromosomes after 10 h culture. In the control groups, a few oocytes stayed at GV or GVBD stage and most reached MI or ATI/MII stage (MI: 44.00 ± 2.35%; ATI/MII: 38.50 ± 1.81%, *n* = 166); while a significant higher rate for MI stage and lower rate for ATI/MII stage were observed in the NIV‐treated groups (MI: 60.90 ± 5.02%, *p* < 0.05; ATI/MII: 13.70 ± 5.23%, *n* = 137, *p* < 0.05; Figure 2C). Further analysis for pro‐MI and MI stage distribution showed that the oocytes exposed to NIV mostly delayed to reach MI compared with the control groups, since the percentage of pro‐MI oocytes was much higher in the NIV‐treated group (Control: 4.16 ± 2.13%, *n* = 166; NIV group: 19.21 ± 3.45%, *n* = 137, *p* < 0.05; Figure 2D). We also measured the distance from the spindle pole to the cortex (length, L) and the diameter (D) of oocytes to check early MI and late MI stage distribution. As shown in Figure 2E, the ratio of the spindle migrated to the cortex (L/D) in the NIV‐treated group was significantly increased compared with the control oocytes (Control: 0.09 ± 0.02, *n* = 43; NIV group: 0.21 ± 0.02, *n* = 47, *p* < 0.01). These results indicated that NIV induced metaphase I block during oocyte meiosis.

### 3.3 NIV affects Cyclin B1 expression for cell cycle progression in oocytes

**FIGURE 3:**
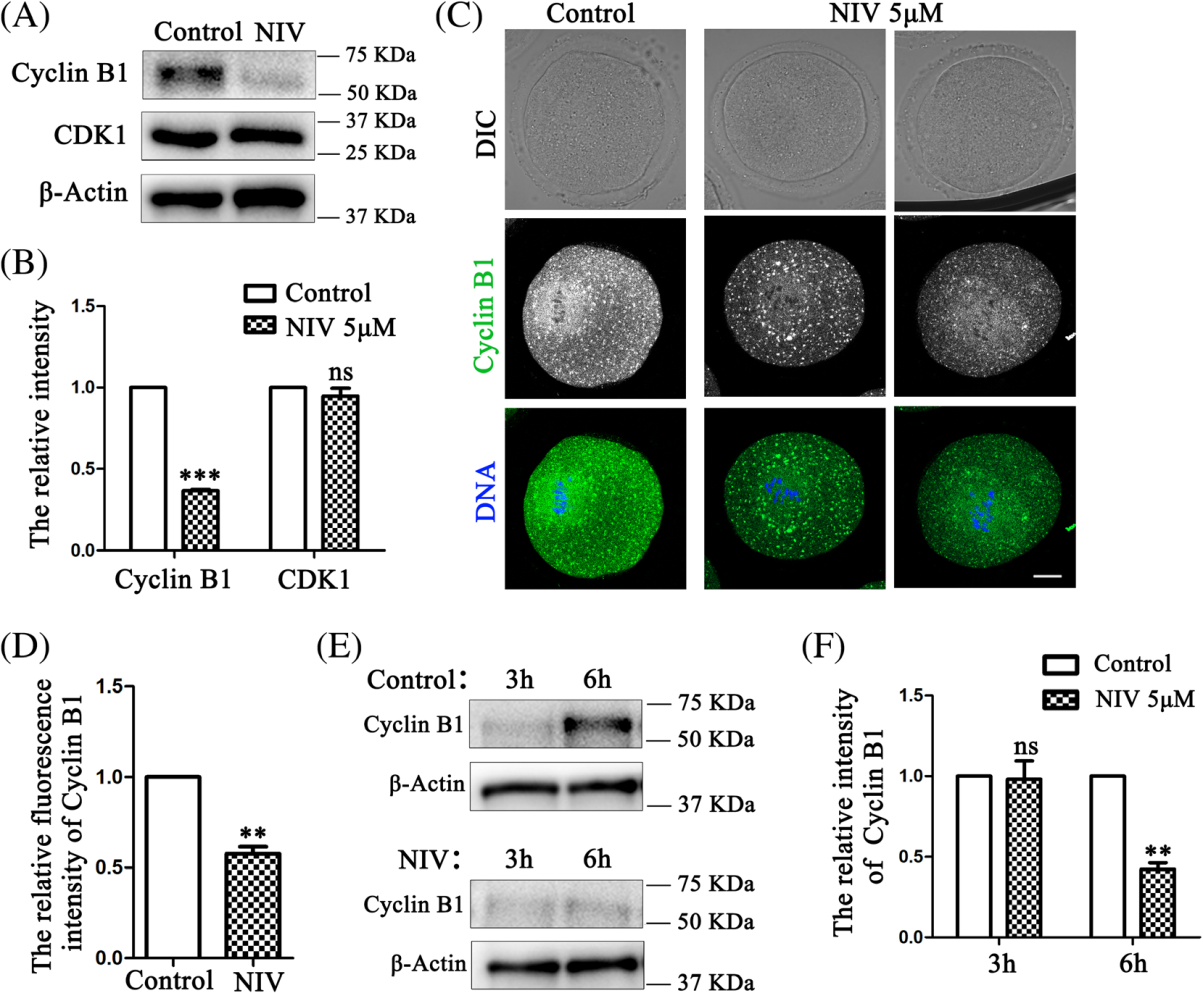
NIV affects Cyclin B1 expression for cell cycle progression in oocytes. (A) Protein levels of Cyclin B1 and CDK1 in oocytes after 9 h culture were determined. A total of 150 oocytes per group were used for detection (A, E). Decreased Cyclin B1 expression and no significant difference of CDK1 expression were observed in the NIV‐treated oocytes. (B) Band intensity analysis showed that the relative intensity of Cyclin B1 was much lower in the NIV treated groups. ***p < 0.001. (C) Exposure to NIV caused Cyclin B1 fluorescence signals significantly weaker than control groups after 9 h culture (Control: *n* = 57; NIV group: *n* = 60). Green, Cyclin B1; Blue, DNA; Bar = 20 μm. (D) The fluorescence intensity of Cyclin B1 was markedly reduced in oocytes with NIV treatment. **p < 0.01. (E) Western blot results for Cyclin B1 expression after 3 h and 6 h culture in the control and NIV‐treated groups. The expression of Cyclin B1 at 6 h culture was decreased in the NIV‐exposed oocytes. (F) Quantitative band analysis of Cyclin B1 expression after oocytes culture to 3 h and 6 h. **p < 0.01.

To explore how NIV induced oocyte cell cycle progression defects, we next examined the activity of MPF, a complex of the catalytic subunit CDK1 and the regulatory subunit Cyclin B1, as it plays a vital role after meiosis resumption. We first quantified the CDK1 and Cyclin B1 level after 9 h culture in NIV‐treated oocytes and found that NIV did not affect the expression of CDK1, but the Cyclin B1 level was dramatically lower than control oocytes (Figure 3A). Statistical analysis showed a significant lower intensity of Cyclin B1 in the NIV exposure oocytes after 9 h culture (Control: 1.00 ± 0.00; NIV group: 0.37 ± 0.01, *p* < 0.001; Figure 3B). We also stained Cyclin B1 and the immunofluorescence results showed that the signals of Cyclin B1 at MI stage were much weaker in the NIV exposure oocytes (Figure 3C). The fluorescence intensity analysis data also confirmed this finding (Control: 1.00 ± 0.00, *n* = 57; NIV group: 0.58 ± 0.04, *n* = 60, *p* < 0.01; Figure 3D). Since Cyclin B1 is rapidly accumulated after GVBD and continuously degraded by an APC/C‐dependent manner before anaphase I onset, we asked whether NIV might induce Cyclin B1 accumulation defect when oocyte re‐enters meiosis. The Cyclin B1 level in the NIV‐treated group after 3 h and 6 h culture was investigated subsequently. Western blotting results showed that the Cyclin B1 expression increased at 6 h culture compared with 3 h in the control group, while it was markedly decreased after NIV treatment in 6 h culture oocytes (Figure 3E), which was confirmed by the band intensity analysis (Control: 1.00 ± 0.00; NIV group: 0.42 ± 0.02, *p* < 0.01; Figure 3F). Therefore, we assumed that NIV affected Cyclin B1 expression for the cell cycle progression of mouse oocytes.

### 3.4 NIV affects microtubule stability in oocyte meiosis

**FIGURE 4:**
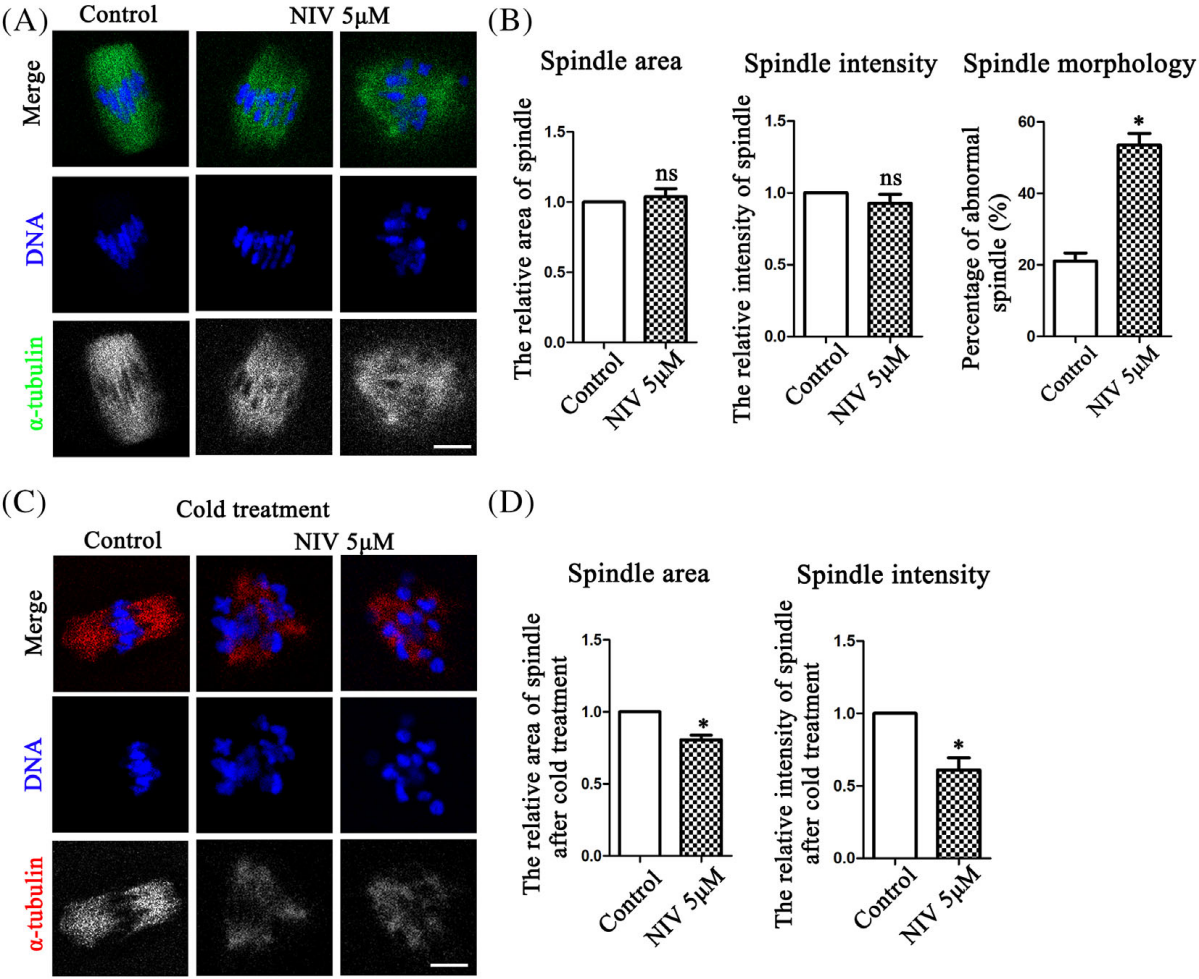
NIV affects microtubule stability in oocyte meiosis. (A) The spindle size and microtubule signals were not affected after NIV exposure compared with the control oocytes, while the spindle morphology of treatment groups was disrupted (Control: *n* = 45; NIV group: *n* = 36). Green, α‐tubulin; Blue, DNA; Bar = 10 μm. (B) The relative fluorescence intensity analysis of microtubule intensity and spindle area calculation showed no significant difference between the control and NIV‐treated groups, while the abnormal rate of spindle morphology was much higher after NIV exposure. ns, *p* > 0.05; *p < 0.05. (C) After 9.5 h culture, the oocytes preformed 5 min cold treatment were used to test the stability of microtubules. After exposure to NIV, the spindle area and microtubule signals were both lower than that in the control groups (Control: *n* = 33; NIV group: *n* = 32). Red, α‐tubulin; Blue, DNA; Bar = 10 μm. (D) Fluorescence intensity analysis showed that spindle area and microtubule intensity in the NIV‐treated group were both lower compared with control group. *p < 0.05.

Microtubules are crucial for cell cycle progression in both mitosis and meiosis, we subsequently explored the effects of NIV on microtubule stability. As shown in Figure 4A, the oocytes in the control groups showed normal two poles and barrel‐shaped spindles after 9.5 h culture, while NIV treatment caused the aberrant spindle morphology. However, there was no significant difference for the spindle size and microtubule intensity between these groups. The spindle area calculation (Control: 1.00 ± 0.00, *n* = 45; NIV group: 1.04 ± 0.06, *n* = 36, *p* > 0.05) and microtubule intensity analysis (Control: 1.00 ± 0.00, *n* = 45; NIV group: 0.93 ± 0.06, *n* = 36, *p* > 0.05) also confirmed this. However, a higher abnormal spindle rate after NIV exposure was found (Control: 21.09 ± 2.22%, *n* = 45; NIV group: 53.50 ± 3.29%, *n* = 36, *p* < 0.05; Figure 4B). While we performed cold treatment to depolymerize the unstable microtubules after 9.5 h culture, we found that the spindle area and microtubule signals were both markedly decreased than the control oocytes (Figure 4C). The area calculation (Control: 1.00 ± 0.00, *n* = 33; NIV group: 0.81 ± 0.03, *n* = 32, *p* < 0.05) and fluorescence intensity analysis (Control: 1.00 ± 0.00, *n* = 33; NIV group: 0.61 ± 0.08, *n* = 32, *p* < 0.05) also confirmed this finding (Figure 4D), indicating that the microtubule stability was destroyed by NIV in oocyte meiosis.

### 3.5 NIV affects K‐MT attachment for SAC in oocyte meiosis

**FIGURE 5:**
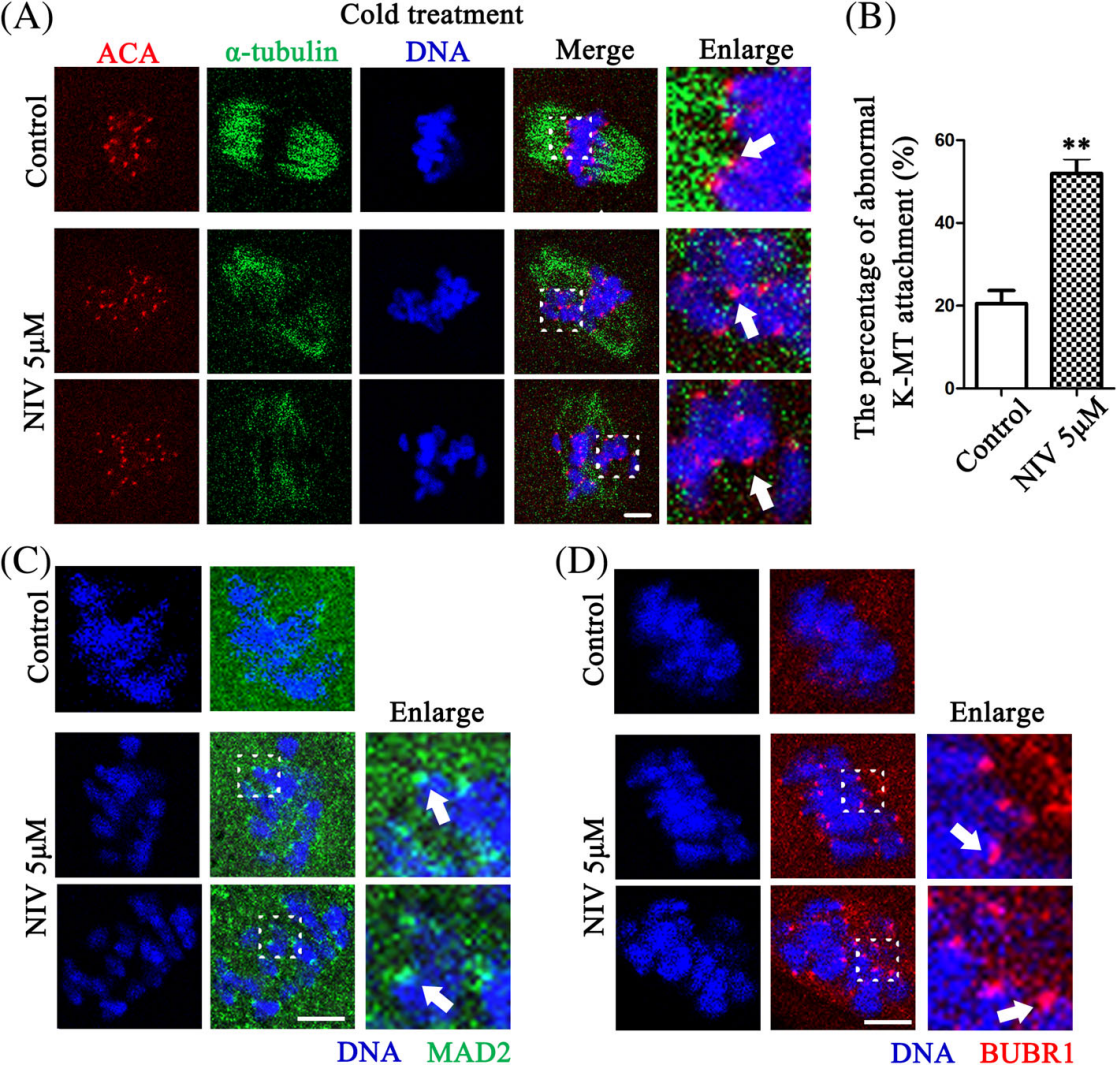
NIV affects K‐MT attachment for SAC in oocyte meiosis. (A) The clear K‐MT stable attachment was observed in the control groups, while there were no microtubules attached to kinetochores after NIV exposure. The arrows highlighted the stable K‐MT attachment in the control and detachment in the treatment oocytes (Control: *n* = 52; NIV group: *n* = 60). Red, ACA; Green, α‐tubulin; Blue, DNA; Bar = 10 μm. (B) The percentage of K‐MT detachment in NIV‐treated oocytes was significantly increased compared with control group. **p < 0.01. (C) Stronger MAD2 fluorescence signals were observed at the kinetochores of the treatment group oocytes, while there were no signals in the control group after 9.5 h culture. The arrows indicated MAD2 signals at the kinetochore. Green, MAD2; Blue, DNA; Bar = 5 μm. (D) Stronger BUBR1 fluorescence signals were observed at the kinetochores of the treatment group oocytes, while there were no signals in the control groups after 9.5 h culture. The arrows indicated BUBR1 signals at the kinetochore. Red, BUBR1; Blue, DNA; Bar = 5 μm.

K‐MT attachment contributes to chromosome alignment and segregation, which depends on microtubule stability. Due to the fact that NIV induced microtubule depolymerization by cold treatment, we presumed that NIV might affect the stabilization of K‐MT attachment. We performed cold treatment to better visualize the stable connection of K‐MT. As expected, exposure to NIV resulted in K‐MT detachment of oocytes, showing with barely microtubules which could attach with kinetochores; while stable microtubules connected with kinetochores in the control oocytes were observed after cold treatment (Figure 5A). The statistical analysis showed that the percentage of abnormal K‐MT attachment was much higher in the NIV exposure group (Control: 20.54 ± 3.13%, *n* = 52; NIV group: 51.99 ± 3.60%, *n* = 60, *p* < 0.01; Figure 5B). Since improper K‐MT attachment signal can activate SAC for MI arrest, we therefore tested the activity/localization of MAD2 and BUBR1, two important regulators of SAC. After culturing oocytes with 9.5 h, strong MAD2 signals were still found at the kinetochores of NIV‐treated oocytes, whereas few signals could be detected in the control oocytes (Figure 5C); a similar result was also observed for the BUBR1 signals (Figure 5D). These findings indicted that NIV caused the continuous SAC activation even after 9.5 h culture, which might be due to K‐MT attachment defect in oocyte meiosis.

## 4 DISCUSSION

**FIGURE 6:**
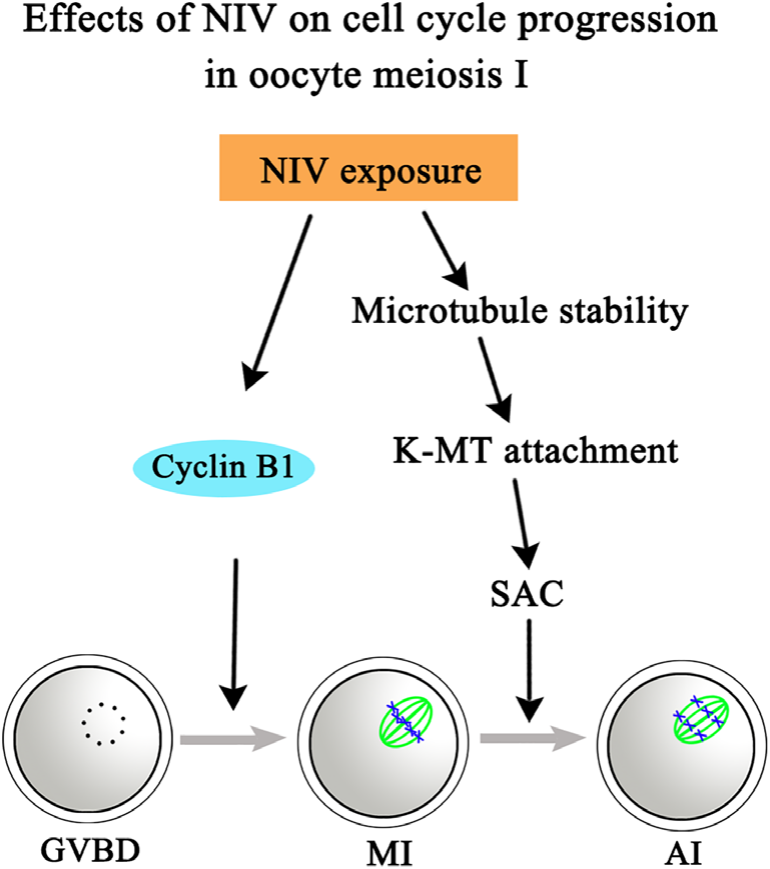
Diagram for the effects of NIV on cell cycle progression in oocyte meiosis. NIV did not affect meiotic resumption but reduced Cyclin B1 expression and activated SAC proteins with unstable microtubules and K‐MT attachment, ultimately led to oocyte meiosis arrest.

Mycotoxins have been attracted great attention since they are widely found worldwide and toxic to humans and animals. Many studies have focused on the oxidative stress, DNA damage and apoptosis to clarify the adverse effects of mycotoxin exposure, but few studies focus on cell cycle aspect. In present study, we used NIV, a typical secondary metabolite of trichothecene mycotoxin, to investigate its effects and mechanisms on cell cycle of oocyte meiosis. Our results indicated that NIV did not affect meiotic resumption, but induced Cyclin B1 expression failure and activated microtubule stability‐dependent SAC proteins which further led to oocyte meiosis arrest (Figure 6).

Exposure to environmental pollutants such as decabromodiphenyl ethane (DBDPE), a brominated flame retardant which is wildly used worldwide, could considerably reduce the oocyte ability to undergo GVBD.^25^ G0/G1 block in J774A.1 cells and G2/M transition failure in HeLa cells after NIV exposure are also observed, implying the adverse effects of NIV on cell cycle progression.^8^, ^9^ In this study we first examined the effects of NIV on oocyte G2/M transition and surprisingly, the results showed that oocyte meiosis resumption was not affected after NIV exposure even with extremely high doses, which was different with other models. To confirm this, we examined the expression of γ‐H2AX which is the marker as the earliest event of DNA damage response,^26^ since environmental harmful effects might induce DNA damage and then activate G2/M checkpoint, showing with the creation of γ‐H2AX.^27^, ^28^ Our results suggested that no change of γ‐H2AX expression after NIV exposure, which confirmed our findings for the negative effects of NIV on oocyte meiosis resumption.

We next analyzed the meiotic progression after GVBD. Our results showed that NIV decreased the oocyte ability to extrude PB1 but it could be rescued after releasing from NIV exposure, which were consistent with previous studies.^13^ Cell cycle analysis indicated that NIV exposure induced pro‐MI or MI stage arrest, suggesting that the harmful effects of NIV on cell cycle control during oocyte meiosis. Cyclin B1, a regulatory subunit of MPF, synthesizes from prophase and associates with CDK1 to form an inactive pre‐MPF before oocyte meiotic resumption. A low level of Cyclin B1 at GV stage and during GVBD is sufficient for mouse oocyte to re‐enter meiosis without *de novo* protein synthesis,^29^ and Cyclin B1 is dramatically high in pro‐metaphase with translation of *Ccnb1* until APC/C activation.^30^ Our results showed low Cyclin B1 level after 3 h culture in both control and treatment groups. Whereas, a significant decreased Cyclin B1 expression was observed after 6 h and 9 h culture in NIV‐exposed groups compared with control groups, indicating that inadequate Cyclin B1 prevented full activation of MPF and ultimately induced oocyte MI arrest. For the mechanism of NIV on Cyclin B1, one possibility is that since trichothecenes could disrupt protein synthesis by associating with 60S subunit of ribosomes,^31^ while NIV is a typical trichothecenes toxin, it is possible that NIV might also inhibit Cyclin B1 expression due to its toxicity on ribosomes. Another possibility is that upon meiosis resumption, intermediate and long 3′‐UTRs forms of *Ccnb1* which repress in quiescent GV oocytes drive Cyclin B1 synthesis with a CPEB1‐dependent manner,^32^ since CPEB1 could inhibit translational activity in the 3′‐UTRs of *Ccnb1*.^33^ Phosphorylated CPEB1 expelled from the repressive components and lead to ribosome loading on *Ccnb1*.^30^ Low level of *Ccnb1* is found in porcine oocytes after exposure to a zinc chelator TPEN, which might underlie the decreased MPF levels and oocyte meiosis arrest.^34^ Whether NIV might interfere these regulatory factors for controlling *Ccnb1* translation or affect the accumulation of maternal mRNAs for low Cyclin B1 level after GVBD need further study.

Besides MPF, the mitogen‐activated protein kinase (MAPK) also plays a crucial role in meiotic cell cycle. MAPK is activated after GVBD and keeps high activity until meiosis II completion.^35^ Extracellular signal‐regulated kinases 1 and 2 (ERK1/2), two of widely reported MAPK family members regulate microtubule organization and spindle assembly.^36^ Recent study shows that there is positive feedback between *Ccnb1* translation and ERK1/2 activation.^37^ Low Cyclin B1 level observed in our results might imply that MAPK signaling pathway disruption, which might explain the reason for the effects of NIV on the spindle. Proper microtubule dynamics is essential for spindle assembly and involves in faithful chromosome segregation. To further investigate the specific mechanism of NIV induced oocyte cell cycle arrest, we consequently explored the effects of NIV on microtubule stability. Our results suggested that NIV significantly destroyed microtubule stability and K‐MT attachment by cold treatment experiment. Correct K‐MT attachment is monitored by SAC proteins which could be activated for MI arrest. MAD2 and BUBR1 are two core components of SAC proteins, which contribute to inhibiting APC/C activity by forming MCC until all chromosomes build bipolar attachment and align at the spindle equator during oocyte maturation.^17^ In our study, positive MAD2 and BUBR1 signals at the kinetochores were found after NIV exposure, demonstrating that NIV activated MAD2/BUBR1‐mediated SAC proteins and ultimately led to oocyte cell cycle arrest.

Taken together, our results indicated that NIV did not affect meiotic resumption but reduced Cyclin B1 expression and activated SAC proteins with unstable microtubules and K‐MT attachment, ultimately led to oocyte meiosis arrest.

## AUTHOR CONTRIBUTIONS

Shao‐Chen Sun, Ping‐Shuang Lu designed the experiments; Ping‐Shuang Lu performed the experiments; Yue Wang, Le Jiao contributed to the materials; Ping‐Shuang Lu analyzed the data. Ping‐Shuang Lu, Shao‐Chen Sun wrote the manuscript. All authors approved the submission of the manuscript.

## FUNDING INFORMATION

This study was supported by the National Key Research and Development Program of China (2021YFC2700100) and the National Natural Science Foundation of China (32170857).

## CONFLICT OF INTEREST

The authors declare no conflict of interest.

## ETHICS STATEMENT

The animals use and experimental programs were carried out in accordance with the guidelines of the Animal Research Committee of Nanjing Agricultural University in China, and this study was specifically approved by the Animal Research Committee of Nanjing Agricultural University.

## Data Availability

All data generated or analysed during this study are included in this published article.
